# Recognition and repair of an incidental umbilical hernia repair during abdominoplasty

**DOI:** 10.1016/j.amsu.2022.104731

**Published:** 2022-09-22

**Authors:** Vladislav Pavlovich Zhitny, Jake Patrick Young, Frank Stile

**Affiliations:** a New York University, Department of Anesthesiology, Perioperative Care and Pain Medicine, New York City, NY, USA; b University of Utah, Department of Biology, Salt Lake City, UT, USA; c Stile Aesthetics, Las Vegas, NV, USA

**Keywords:** Hernia repair, Abdominoplasty, Plastic surgery

## Abstract

**Introduction:**

Umbilical hernias are found in 2% of the American adult population with increasing prevalence in overweight and multiparous women. A mesh repair is considered to be a suitable option for those desiring non-cosmetic surgical repair. Despite the mesh plug's reported value in reduction of the recurrence of umbilical hernia from 11% to 1%, there is an increased risk in de-vascularizing the umbilicus with its use.

**Presentation of Case:**

We present a case which avoids fascial incisions near the umbilicus, thus preserving the blood supply employing sutures to reduce the small abdominal wall defect which is then further reinforced by overlying rectus muscle plication.

**Discussion of Case:**

Hernia repair can be associated with a host of issues, minor and major, including regional tissue ischemia and the distortion of natural anatomy, likely due to ischemia of the epigastric vessels. Abdominoplasty is a suitable option for patients with redundancy of the abdominal skin and laxity abdominal wall musculature. Abdominoplasty has excellent exposure and correction of abdominal wall hernias. This “anatomic repair” employing sutures to reduce the small abdominal wall defect which is then further reinforced by overlying rectus muscle plication can be used in place of a mesh overlay for the purposes of umbilical hernia repair especially when the hernia may be asymptomatic.

**Conclusion:**

Abdominoplasty uniquely allows for an open hernia repair when anticipated or discovered at the time of surgery and is considered a true anatomical repair of an umbilical hernia which does not necessitates the use of foreign materials.

## Highlights

• Umbilical hernias often remain undiscovered on physical exam prior to abdominoplasty.
• Special care should be taken around area of an umbilicus during an abdominoplasty due to risk of hernia perforation and umbilical devascularization.
• Abdominoplasty should be recommended over panniculectomy for patients with a known history of an umbilical hernia.
• Abdominoplasty should be considered in the treatment of umbilical hernias when weighted against an umbilical hernia repair using a mesh.

## 1 Introduction

Abdominoplasty is currently the fifth-most commonly requested cosmetic operation in the United States [1,2]. Currently, abdominoplasty functions to correct abdominal skin redundancy and fascial laxity, which commonly presents with pregnancy, increased weight, advanced age or any combination of these.

Abdominal wall hernias are more common in overweight and multiparous women. They are often diagnosed by clinical history and/or physical exam [3]. Pregnancy may cause herniation or make a preexisting hernia more detectable due to elevated intra-abdominal pressure. In infants, these hernias are typically congenital. Umbilical hernias in adults occur subsequent to the closure of the umbilical ring and are due to a progressive weakening of the cicatricial tissue closing the ring [4,5]. Umbilical hernias may also be discovered incidentally during an abdominoplasty operation. Correction of the hernia can also improve the overall abdominal appearance. Concerns about an incidental hernia not detected prior to procedure include possibility of perforation.

Many surgeons opt for a two-part procedure instead of simultaneous hernia repair and abdominoplasty [5,6]. When completed simultaneously, the current literature describes that there is the potential to de-vascularize the umbilicus when hernia repair and abdominoplasty are performed together, circumferential incisions around the umbilicus to reduce the hernia can result in separation of the umbilicus from the flap, and thus compromise the umbilical blood supply as it relates to skin necrosis however it does not outline the technique from the stand point of incidental hernia recognition and repair [[4], [5], [6]]. For this reason, patients with a known history of an umbilical hernia are often encouraged to undergo abdominoplasty over a panniculectomy repair. Yet another technique is that of surgical mesh repair. Abdominoplasty also allows for a procedure that repairs the defect without use of foreign materials. Surgeons should provide the patient risks and benefits of both surgeries based on patient's comorbidities and preference. We present a case which avoids fascial incisions near the umbilicus, thus preserving the blood supply and describes an exact technique that addresses an incidental umbilical hernia recognition and repair with proper care.

## 2 Presentation of Case

**Fig. 1:**
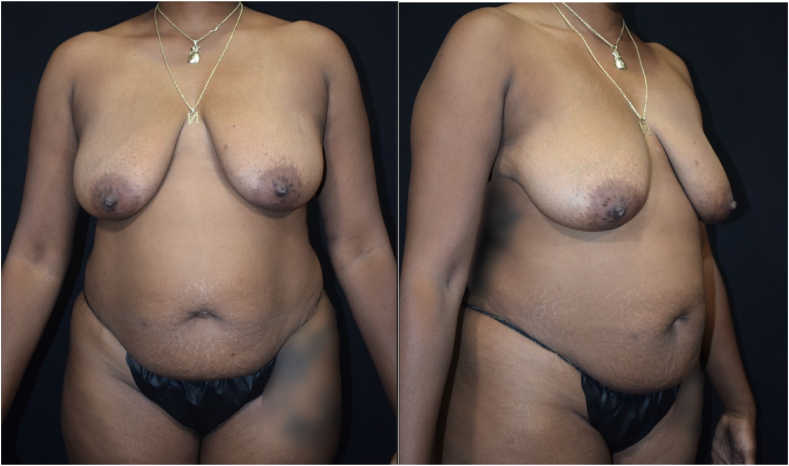
Hidden umbilical hernia, no appreciable mass on pre-operative physical exam.

The patient was a 38-year-old G4 P4004 female who presented for abdominoplasty and diastasis recti repair. Physical exam pre-operatively in the clinic did not demonstrate any gross deformities or masses **[**Fig. 1**]**. The patient furthermore had not complained of any symptoms of bloating or abdominal pain. Vitals were normal pre-operatively and BMI was 27.4. The patient denied any prior surgeries and personal medical history was non-contributory other than a history of smoking tobacco (2 packs a day) and cannabis use.

**Fig. 2:**
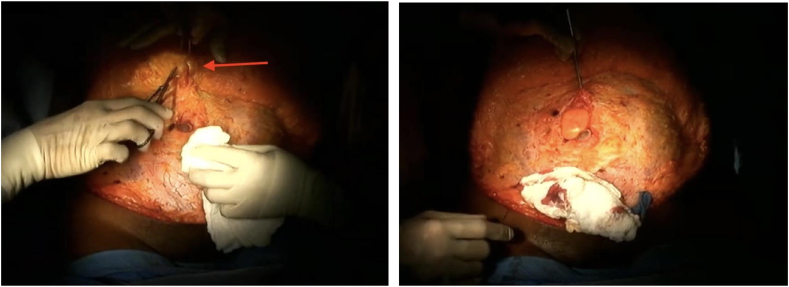
A) Large incidental hernia discovered at abdominoplasty. B) Fully exposed umbilical hernia. Approximately 2.0 cm hernia ring with intra-abdominal fat protruding.

The patient was marked in the pre-operative holding area and her abdomen was prepped and draped in sterile fashion from the chest to the mid-thighs in the operating room. Once the abdominal wall fascia was reached, dissection of the abdominal fat from the fascia proceeded superiorly and laterally to the level of the umbilicus. Upon reaching the umbilicus, it was discovered that patient had an umbilical hernia, evidenced by an almost 2.0cm rent in the fascia with what appeared to be omental fat exiting from its normal intra-abdominal location **[**Fig. 2**]**.

Great care was taken to isolate the herniated intraabdominal contents. Using both blunt and sharp dissection techniques. Once our dissection progressed beyond the hernia, the remainder of the skin and fat abdominal flap was further elevated superiorly to the level of the xyphoid centrally and the lower coastal margins bilaterally.

We returned our attention to the umbilical hernia. Great care was taken to identify and isolate the hernia ring and both blunt and sharp dissection techniques were used once again to separate the herniating omental fat from the hernia ring. This is sometimes a difficult and tedious due to the adhesive process that may result from chronic inflammation. We were always mindful to behave cautiously because it is not always easy to rule out small or large intestine that may be included in the herniated structures. It is important to note that a typical hernia sac is not always encountered in these hernias.

The freely reducible herniated structures are now returned to their intraabdominal position. The rent is closed directly using a 2–0 Proline suture. This closure will be reinforced by the eventual reduction of this patient's diastasis which is part of a standard abdominoplasty procedure. For all intents and purposes, this can be considered a true anatomical repair of an umbilical hernia which does not necessitates the use of foreign materials such as mesh which associated with a host of their own challenges.

**Fig. 3:**
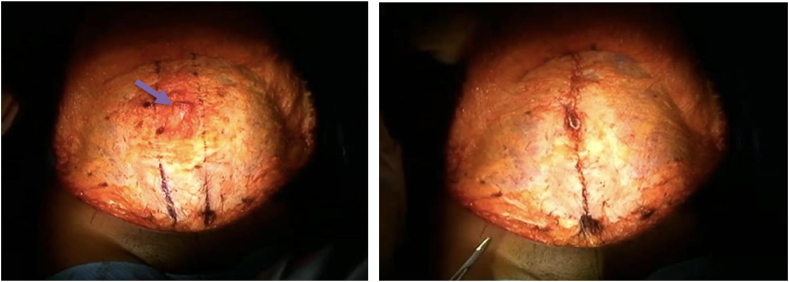
A) Arrow indicates location of repaired rent in fascia. Vertical blue ink lines demonstrate medial rectus edges for planned rectus diastasis repair B) Photo shows abdominal wall after reduction of the diastasis using our described technique. (For interpretation of the references to colour in this figure legend, the reader is referred to the Web version of this article.)

With the hernia reduced and the hernia ring closed, we next continue with our standard abdominoplasty procedure as mentioned earlier. We choose to use 2-0 double-stranded-looped Neurolon suture to reduce the diastasis in all my abdominoplasty procedures. Beginning at the Xyphoid, we reduce the diastasis using a continuous suture technique with sutures placed at 1cm intervals employing an imbricating technique grabbing approximately 1cm of fascia on either side of the diastasis. This suture is tied at the upper side of the umbilical stalk. The exact technique is used beginning once again at the lower side of the umbilical stalk continuing down to approximately the pubic tubercle **[**Fig. 3**]**.

With the diastasis reduced and the hernia repaired, the redundant skin and fat of the abdominal flap is now excised. Our freely mobile skin flap was carefully drawn inferiorly, the wound edges are closed, and the umbilicus is brought out through a new opening. The patient recovered well with no complications following completion of the procedure. The patient was lost to follow-up.

## 3 Discussion

Umbilical hernias are found in 2% of the American adult population, but the prevalence is increased among obese and multiparous women [7]. Occasionally they are an incidental finding during abdominoplasty procedures, but prior studies have cautioned the safety of these procedures and preservation of the umbilicus when abdominoplasty is combined with other abdominal procedures [2]. Furthermore, hernias can be associated with a host of issues, minor and major, including regional tissue ischemia and the distortion of natural anatomy, likely due to ischemia of the epigastric vessels. Other associated risks include pulmonary embolism and hemorrhage. Hernia repair cannot always be planned prior to procedure due to lack of physical signs or personal history including – masses, abdominal pain, bloating etc.

After performing over 2600 abdominoplasty procedures and using the described technique to reduce numerous umbilical and other hernias that have been incidentally discovered during these procedures, we have yet to find a detectable recurrence of these hernias in our patients. Despite the mesh plug's reported value in reduction of the recurrence of umbilical hernia from 11% to 1%, there is an increased risk in de-vascularizing the umbilicus with its use [8,9]. Our team elected to use only sutures to fix the defect. However, literature elsewhere has also recommended a mesh overlay to reduce recurrence [2,8,9].

Panniculectomy and abdominoplasty are both procedures which are used to contour the lower abdomen [[10], [11], [12]]. A panniculectomy is a more conservative procedure and is used for function restoration which may be impaired by large pendulous pannus. Abdominoplasty, however, not only removes excess adipose tissue and skin, but also tightens the musculature and relocates the umbilicus. Abdominoplasty is considered a cosmetic procedure.

The anatomical limitations of panniculectomy, with respect to extent of dissection may mean that hernias are not identified and therefore not repaired. The diastasis is also not addressed during a panniculectomy. Frequently confused with hernia, diastasis of the rectus muscles refers to the separation of the midline muscles, which is especially common after pregnancy [10]. Women often have a more severe diastasis below the umbilicus. With diastasis, the fascia remains intact, preventing any strangulation or incarceration of the bowel.

Abdominoplasty uniquely allows for an open hernia repair when anticipated or discovered at the time of surgery. Abdominoplasty is a suitable option for patients with redundancy of the abdominal skin and laxity abdominal wall musculature. Abdominoplasty always allows for excellent exposure and correction of abdominal wall hernias. This “anatomic repair” employing sutures to reduce the small abdominal wall defect which is then further reinforced by overlying rectus muscle plication can be used in place of a mesh overlay for the purposes of umbilical hernia repair especially when the hernia may be asymptomatic.

## Ethical approval

This article type (case report) does not require a formal ethical committee approval.

## Sources of funding

No Source of Funding.

## Author contribution

All authors contributed to data collection, drafting the article, revising the article, gave approval of the version to be published, and agreed to be accountable for all aspects of the work.

## Consent

Written informed consent was obtained from the patient for publication of this case report and accompanying images. A copy of the written consent is available for review by the Editor-in-Chief of this journal on request.

## Registration of research studies

Not required for case report.

## Guarantor

Mr. Jake Patrick Young.

University of Utah.

Department of Biology, University of Utah, Salt Lake City, UT.

257 1400 E.

Salt Lake City, Utah, 84112, USA Email: U1356315@utah.edu.

## Consent of patient

Written informed consent was obtained from the patient for publication of this case report and accompanying images. A copy of the written consent is available for review by the Editor-in-Chief of this journal on request.

## Peer review

Provenance and peer review.

Not commissioned, externally peer-reviewed.

## Declaration of competing interest

The authors have no competing interests to declare.
